# Monensin phase-out in Norwegian turkey production decreases Bifidobacterium spp. abundance while enhancing microbial diversity

**DOI:** 10.1099/mgen.0.001466

**Published:** 2025-08-20

**Authors:** Håkon Pedersen Kaspersen, Eva Lena Estensmo, Jannice Schau Slettemeås, Thomas H. A. Haverkamp, Siri Kulberg Sjurseth, Silje Granstad, Camilla Sekse, Rikki Franklin Frederiksen, Anne Margrete Urdahl

**Affiliations:** 1 Norwegian Veterinary Institute, Ås, Norway; 2 Nortura SA, Oslo, Norway

**Keywords:** antibiotics, *Bifidobacterium*, diversity, microbiota, monensin, turkey

## Abstract

Intestinal tissue damage caused by coccidiosis is an important predisposing factor for necrotic enteritis in turkeys, and both diseases are common health issues in turkey production. In Norway, the in-feed ionophore coccidiostat monensin has been used as a preventive measure to combat coccidiosis since the late 1980s. In 2022, however, preventive use of monensin was phased out, which led to an undesired increase in antibiotic treatments among turkey flocks, largely due to necrotic enteritis. The aim of this study was to investigate the overall effects of the preventive monensin use and antibiotic treatment on the turkey caecal microbiota. A total of 102 flock samples from the Norwegian turkey population were included, and metagenomic datasets were generated through shotgun sequencing. All datasets were processed with the Taxprofiler pipeline, followed by diversity, redundancy and differential abundance analyses in R. A significant decrease in alpha and beta diversity was observed for the caecal samples from turkeys exposed to monensin, compared with the non-exposed. An increased abundance of *Bifidobacterium* spp. was observed in the samples from monensin-exposed turkeys, including *Bifidobacterium pullorum*,* Bifidobacterium longum*,* Bifidobacterium pseudolongum*,* Bifidobacterium pseudocatenulatum* and *Bifidobacterium animalis*. Additionally, a decrease in *Megamonas* and *Megasphaera* species was detected in these samples. Further, species within the *Clostridium* genus were higher in abundance among the samples from female turkeys compared with male turkeys. The results indicate that the use of monensin seems to decrease the overall diversity and promote the abundance of *Bifidobacterium* spp. in the caecum of turkeys, while decreasing the abundance of *Megamonas* and *Megasphaera* species. The use of monensin may be beneficial for the gut microbiota due to an increase in favourable *Bifidobacterium* spp. In contrast, treatment with phenoxymethylpenicillin (penicillin V) early in the turkey life cycle does not seem to cause long-term changes in the caecal microbiota composition. However, further studies are needed to investigate the effects of a decreased abundance of *Bifidobacterium* spp. and increased gut microbiota diversity in turkeys in the absence of monensin use.

## Data Summary

All reads have been uploaded to the SRA under the project PRJNA1145617. All R scripts used for data analysis and figures are available at https://github.com/NorwegianVeterinaryInstitute/TurkeyBiom.

**Impact Statement**

The ionophore coccidiostat monensin has been used as a prophylactic measure against coccidiosis in the Norwegian turkey production up until 2022. The effect of monensin on the gut microbiota has not been extensively studied, and the samples from 2022 allowed us to characterize the gut microbiota of flocks administered monensin and flocks administered monensin and penicillin V, compared with flocks where neither were administered. We utilized deep shotgun sequencing to characterize the caecal microbiome. We identified that the use of monensin leads to a higher abundance of *Bifidobacterium* spp. and reduced abundance of *Megamonas* and *Megasphaera* species, but also an overall decrease in diversity. We identified that treatment with antimicrobials earlier in the life cycle of the turkeys does not seem to affect the microbiota composition when sampling at slaughter. Additionally, we argue that the reduced abundance of *Megamonas* and *Megasphaera* is likely related to altered competition dynamics rather than a direct antibacterial activity of monensin. Identifying how the host microbiota is affected by various treatments is important from an animal welfare perspective, and the result from this study provides insight into how monensin use affects the microbiota composition in turkeys, which in turn may affect how the poultry industry utilize monensin in other countries.

## Introduction

Turkey meat represents a relatively small segment of the Norwegian poultry market, which is primarily dominated by chicken production. Aside from one small organic farm, all turkey meat in Norway is produced by a single company, which slaughters approximately one million birds per year. The turkey breed used is B.U.T. premium, and the parent stock is imported as day-old poults from Great Britain or France [1]. In 2022, there were 43 turkey producers in Norway, and a total of 995,876 turkey poults were hatched [2]. The turkey farms are primarily concentrated in a limited area of Eastern Norway, near the hatchery and slaughterhouse, highlighting the regional focus of this specialized sector within the broader poultry industry. Turkey farming in Norway is primarily conducted in indoor systems, where birds are raised in controlled environments, including regulated temperature, ventilation and lighting. Male and female turkeys in fattening flocks are kept separate, although there are no biosecurity barriers between the sexes. Females and males are slaughtered at 84–91 and 126–133 days, respectively.

The most common health issues in turkey production in Norway are gastrointestinal disorders including coccidiosis, necrotic enteritis and gizzard erosion and ulceration syndrome (GEU). In some cases, necrotic enteritis and GEU may require antibiotic treatment. The use of antibiotics in Norwegian food-producing animals is strictly regulated and subject to mandatory reporting. Hence, antibiotics are only used for therapeutic purposes, and in the case of turkeys, this is restricted to clinical cases of GEU (typically at the age of 10–28 days) and/or necrotic enteritis with increased mortality (typically at the age of 28–56 days). Phenoxymethylpenicillin, also known as penicillin V (PcV), is the only antibacterial substance used and is administered through drinking water. In most cases, the entire flock is treated; however, in houses with separate drinker lines for male and female turkeys, only the affected sex is treated. The daily dosage is determined by the practising veterinarian, typically 50 mg of PcV per kg of live weight. In peracute cases with high mortality, a daily dosage of up to 68 mg of PcV per kilogramme of live weight might be used for the initial 2–3 days. All cases are treated for 5 days, and the birds have continuous access to medicated drinking water throughout the treatment period.

In Norwegian turkey production, the focus is on preventive measures rather than treatment, primarily through biosecurity and proper management routines, to maintain overall flock health and minimize the need for antibiotic interventions. Until 2022, the ionophore coccidiostat monensin was used as a prophylactic measure against coccidiosis. Since the intestinal damage associated with coccidiosis can predispose birds to necrotic enteritis, and monensin is effective against both coccidia and Gram-positive bacteria such as *Clostridium perfringens*, it is likely that monensin also contributed to the control of both necrotic enteritis and GEU in turkeys [3]. In grow-outs with monensin as an in-feed anticoccidial drug, monensin was used continuously from the day of hatch until 6–9 weeks of age, typically around 8 weeks of age. No anticoccidial vaccines or antibiotic growth promoters were used [4]. Monensin was phased out of the turkey production in 2022, encouraged by the phase-out of narasin in broilers in 2015.

Several studies have characterized the general caecal and/or ileal microbiota of turkeys [5,10]. However, there are limited studies that have looked into the effect of monensin on the overall microbiota [11,12]. Generally, most studies have utilized 16S rRNA gene sequencing rather than shotgun sequencing. According to these studies, the bacterial composition of the caecal microbiota of turkeys is predominantly comprised of phyla such as *Bacillota*, *Bacteroidota*, *Actinomycetota* and *Pseudomonadota*. Within these phyla, specific genera such as *Clostridium*, *Ruminococcus*, *Lactobacillus* and *Bacteroides* are consistently prominent in the caecal microbiota.

The aim of this study was to characterize the caecal microbiota of healthy turkeys sampled at slaughter and to investigate how monensin use and antimicrobial treatment alter the caecal microbiota.

## Methods

### Sample material and metadata

The samples were retrieved through the Norwegian surveillance programme for antimicrobial resistance in feed, food and animals in 2022 (NORM-VET) [13]. At slaughter, all fattening turkey flocks processed in Norway in 2022 (*n*=110) were sampled. Individual caeca were sent on ice to the Norwegian Veterinary Institute (NVI) overnight. Upon arrival at the NVI, caecal materials from the individual samples were homogenized into one flock sample, each comprised of ten individual ceca, and flash-freezed by transferring an aliquot of 0.25–0.5 g per sample into a 1 ml LVL tube and lowering it in liquid nitrogen for about 4 min. The frozen tube was immediately transferred to a −80 °C freezer for long-term storage. The 110 samples originated from a total of 40 different farms, of which 38 farms had more than 1 turkey flock slaughtered in the sampling period. Of the 110 samples, 8 were excluded as they originated from a parent flock (*n*=1) and organic farms (*n*=2) or were duplicates of the same flock (*n*=5). Of the remaining 102 samples (Table 1), 25 (24.5 %) were from males and 77 (75.5%) from females, originating from a total of 38 farms with an average of 2.7 flocks per farm. The average age of slaughter and sampling for males and females was 131.6 and 89.5 days, respectively. In total, 4 (3.9%) of the flocks had been treated with PcV and exposed to monensin, 15 (14.7%) were only exposed to monensin, 36 (35.3%) were treated with PcV without being exposed to monensin and 47 (46.1%) were neither exposed to monensin nor treated with PcV. Of the 40 flocks treated with antibiotics, 32 (80.0%) were treated once, while 8 (20.0%) were treated twice. In Norway, turkey feed is supplied by different feed mills and varies in composition but is generally formulated to meet the nutritional requirements of the hybrid used.

**Table 1.** Overview of caecal samples from 102 Norwegian turkey flocks sampled in 2022

Sex	Average age (sd)	Exposure group	*n*	Per cent (%) group	Per cent (%) all
Female	89.5 (2.8)	M+P	3	3.90	2.94
		M	8	10.39	7.84
		P	29	37.66	28.43
		None	37	48.05	36.27
	Sum female		77	100	75.49
Male	131.6 (2.8)	M+P	1	4.00	0.98
		M	7	28.00	6.86
		P	7	28.00	6.86
		None	10	40.00	9.80
	Sum male		25	100	24.51
Sum all			102		

The table presents all the samples included in this study, stratified on sex and exposure groups. M+P, monensin and phenoxymethylpenicillin (PcV); M, monensin only; P, PcV only; None, neither monensin nor PcV.

### DNA extraction and sequencing

The samples were thawed at room temperature, and DNA was extracted with the QIAamp PowerFecal Pro DNA Kit (Qiagen, Hilden, Germany), following the manufacturer’s recommendations and with an automated protocol on the QIAcube connect robot (Qiagen). The samples were prepared by adding 250 mg of the thawed caecum content into PowerBead Pro tubes (Qiagen). Then, 800 µl CD1 buffer was added to each tube, before the samples were homogenized using FastPrep-24™ 5G (MP Biomedicals) in 3 rounds × 60 s at 5 m s^−1^ with 5-min rest between the rounds. The samples were centrifuged at 15,000 ***g*** for 1 min, and 500 µl of the supernatant was transferred to the centre of the rotor adapter. The samples were then prepared and extracted according to the PowerFecal DNA Pro IRT protocol on the QIAcube and eluted in 100 µl. The concentration of the eluted DNA was measured by the Qubit BR HS assay (Thermo Fisher Scientific, Waltham, MA, USA) and by Nanodrop (Thermo Scientific™, MA, USA) by following the manufacturer’s recommendations and stored at −20 °C. Extraction blanks and the ZymoBIOMICS microbial community standard II (Zymo Research, Irvine, CA, USA) were used as sequencing controls and extracted in the same manner as described above.

All sample DNA extracts were diluted 1 : 10, and both controls and diluted samples were sent to the Norwegian Sequencing Centre for Illumina DNA library preparation (Illumina, San Diego, CA, USA) and sequencing on 3/4 of a NovaSeq S4 flowcell (Illumina) to at least 50 million 150 bp paired-end reads per sample.

### Bioinformatic analysis

#### Quality control and taxonomic classification

The Taxprofiler pipeline [14,15] v.1.1.4 was used to run basic quality control, read filtering and taxonomic classification. Briefly, reads were trimmed and adapters were removed, using a minimum read length size of 36 bp, followed by complexity filtering. All reads mapping to the turkey genome (*Meleagris gallopavo*, GCF_000146605.3), phiX (NC_001422) and the human reference genome v.19 [16] were removed. Reads from several runs were merged before taxonomic classification with Kraken2 [17] v.2.1.2 and Bracken [18] v.2.7, using the pre-built plusPFP database from the AWS cloud (updated 5 June 2023) with a read length of 150 bp. Kraken-biom [19] v.1.2.0 was used to generate a biom file from the corrected Kraken2 reports from Bracken.

#### Statistical analysis

The biom file was imported into R v.4.3.0 using phyloseq [20] v.1.44.0. Read counts were rarefied to the sample with the lowest number of reads. For ANOVA, the following model was used:


Y ∼\ exposure\ group+host\ sex+farm\ ID

where ‘exposure group’ equals the four exposure groups described in Table 1, host sex equals the sex of the sampled birds and farm ID equals the ID number of the farm where the birds were reared. For all statistical tests, *P*-values <0.05 were considered significant. When estimating differences between groups at the phylum level, rarefied counts were transformed with the centre log ratio method before ANOVA was applied.

The Shannon diversity index (alpha diversity) was calculated using the ‘estimate_richness’ function from phyloseq. ANOVA was used to estimate the effect of the different variables on the alpha diversity, using the model described above. A Tukey Honest Significant Difference (HSD) test was applied to identify which pairs were significant.

Beta diversity (Bray–Curtis) was calculated using the ‘distance’ function from phyloseq. Significant differences in beta diversity were estimated with a permutational multivariate ANOVA (PERMANOVA), implemented in the adonis2 function from vegan [21] v.2.6.4 with 9,999 permutations, using the same model as described above. A pairwise PERMANOVA was used to identify significant pairs. Dispersion was estimated with the betadisper function using the spatial median method.

Redundancy analysis (RDA) was used to explore the correlation between the bacterial microbiota composition on the genus level and the different variables, e.g. exposure group, host sex and farm ID. The RDA was run using the capscale function from vegan with square root transformation of the Bray–Curtis distances. Only genera that were present in at least 1% abundance were included, using the same model as described above. Collinearity was investigated with the ‘vif.cca’ function. ANOVA was used to determine the significance of each factor variable within the model. Variables were fitted to the model using the ‘envfit’ function with 999 permutations. The top 15 significant genera, ordered by their *R*-value, were plotted.

Lastly, DESeq2 [22] v.1.40.2 was used to identify differentially abundant species with the median-of-ratios method and Wald tests, using the same model as described above. The value ‘none’ was set as a reference within the ‘group’ variable. Only taxa with a relative abundance of at least 1% were included in the analysis.

## Results

### Read depth and taxonomic classification

The average read depth after sequencing all samples was 81.8 million (sd 45.2), with a range of 49.6 to 334.5 million reads. On average, 59.5% (sd 3.34%) of the trimmed and filtered reads were unclassified by Kraken2. A total of 32 extremely low-abundant taxa (0–19 reads) were excluded after rarefaction normalization due to zero counts in all samples. The negative control and mock community were investigated to verify the sequencing result and the level of contamination.

### Relative abundance of taxa

**Fig. 1.**
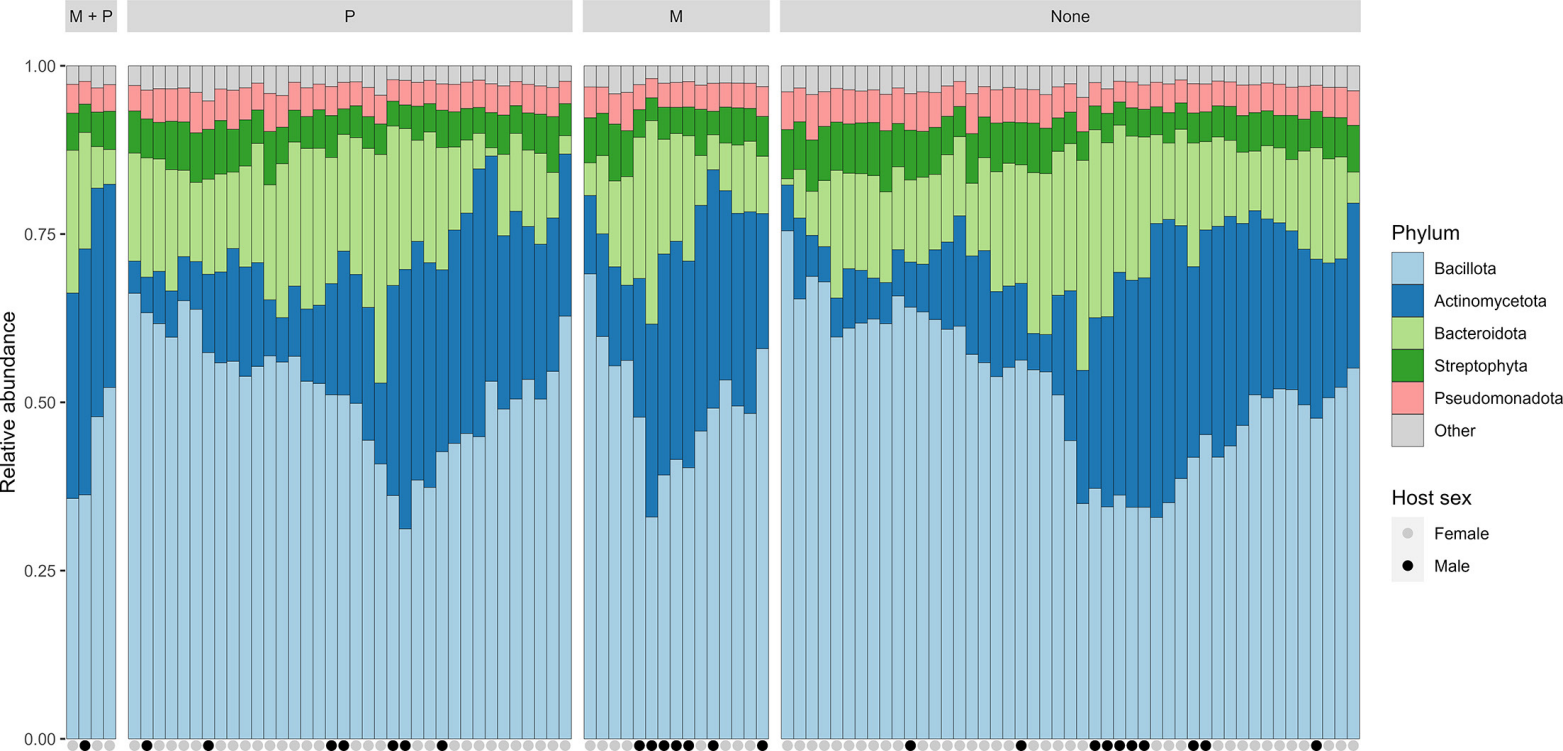
Relative abundance of the top 5 phyla identified in pooled caecal samples from 102 Norwegian turkey flocks. Figure is stratified on exposure groups: M+P, monensin and phenoxymethylpenicillin (PcV); M, monensin only; P, PcV only; and None, neither monensin nor PcV. Grey and black circles below the x-axis represent the sex of the birds from which the sample was derived.

Bacteria were identified as the major taxonomic kingdom in all samples, with an average relative abundance of 93.1 % (sd 1.6%), followed by Eukaryota (6.4%, sd 1.5%), Archaea (0.36%, sd 0.24%) and Viruses (0.11%, sd 0.08%). Major phyla across all kingdoms included *Bacillota* (51.2%, sd 9.87%), *Actinomycetota* (20.7%, sd 10.72%), *Bacteroidota* (15.0%, sd 6.35%), *Streptophyta* (5.7%, sd 1.35%) and *Pseudomonadota* (4.4%, sd 0.73%) (see Fig. 1).

One-way ANOVA revealed a significant difference in the major phylum abundance between the four exposure groups [*F*(12,300)=2.44, *P≤*0.01], host sex [*F*(4,300)=11.11, *P*<0.01] and farm ID [*F*(148,300)=1.40, *P*<0.01], with effect sizes of *η*^2^=0.011, *η*^2^=0.013 and *η*^2^=0.066, respectively. The post hoc Tukey test revealed differences between the sexes for the phyla *Actinomycetota* and *Bacteroidota* (*P*<0.05, Table 2) and for the phylum *Actinomycetota* between the exposure group M+P and M against the ‘None’ group (*P*<0.05). Additionally, differences in the abundance of *Actinomycetota* and *Bacteroidota* were detected between farms.

**Table 2.** Significant differences in major phylum abundance between farms, host sex and exposure groups in pooled caecal samples from 102 Norwegian turkey flocks, tested with one-way ANOVA and a post hoc Tukey HSD test

Phylum	Group 1	Group 2	Tukey *P*-value
*Actinomycetota*	Male	Female	0.0006
	None	M+P	0.0341
	None	M	0.0104
	Farm 23	Farm 13	0.0089
	Farm 28	Farm 13	0.0082
	Farm 30	Farm 13	0.0329
	Farm 37	Farm 13	0.0195
	Farm 9	Farm 13	0.0014
	Farm 23	Farm 15	0.0353
	Farm 9	Farm 15	0.0055
	Farm 28	Farm 15	0.0330
	Farm 9	Farm 26	0.0288
*Bacteroidota*	Male	Female	0.0000
	Farm 18	Farm 14	0.0497

Exposure groups: M+P, monensin and phenoxymethylpenicillin (PcV); P, PcV only; None, neither monensin nor PcV.

On the genus level, *Bifidobacterium* (12.9%, sd: 9.75%) was on average the most abundant genus, followed by *Faecalibacterium* (8.9%, sd: 3.22%), *Subdoligranulum* (6.0%, sd: 1.68%), *Alistipes* (6.0%, sd: 3.97%), *Megamonas* (3.2%, sd: 2.51%), *Bacteroides* (2.7%, sd: 1.49%) and *Barnesiella* (1.4%, sd: 2.79%) (Table 3).

**Table 3.** Average relative abundance (per cent) and sd (in brackets) of the top 5 genera in each exposure group of pooled caecal samples from 102 Norwegian turkey flocks

	Average relative abundance (sd)				
**Genus**	**M+P**	**P**	**M**	**None**	**All samples**
*Bifidobacterium*	24.6 (5.1)	11.4 (9.21)	18.7 (9.19)	11.1 (9.43)	12.9 (9.75)
*Faecalibacterium*	8.8 (4.08)	8.5 (2.81)	9.1 (2.87)	9.3 (3.59)	8.9 (3.22)
*Subdoligranulum*	6.0 (1.12)	5.6 (1.73)	6.5 (2.09)	6.2 (1.49)	6.0 (1.68)
*Alistipes*	4.3 (3.45)	6.6 (4.18)	4.5 (4.25)	6.1 (3.72)	6.0 (3.97)
*Megamonas*	0.0	4.1 (2.59)	0.0	3.5 (2.12)	3.2 (2.51)
*Bacteroides*	0.0	0.0	2.9 (1.6)	0.0	2.7 (1.49)
*Barnesiella*	3.8 (4.22)	0.0	0.0	0.0	1.4 (2.79)

Exposure groups: M+P, monensin and phenoxymethylpenicillin (PcV); P, PcV only; M, monensin only; None, neither monensin nor PcV.

### Diversity

#### Alpha diversity

**Fig. 2.**
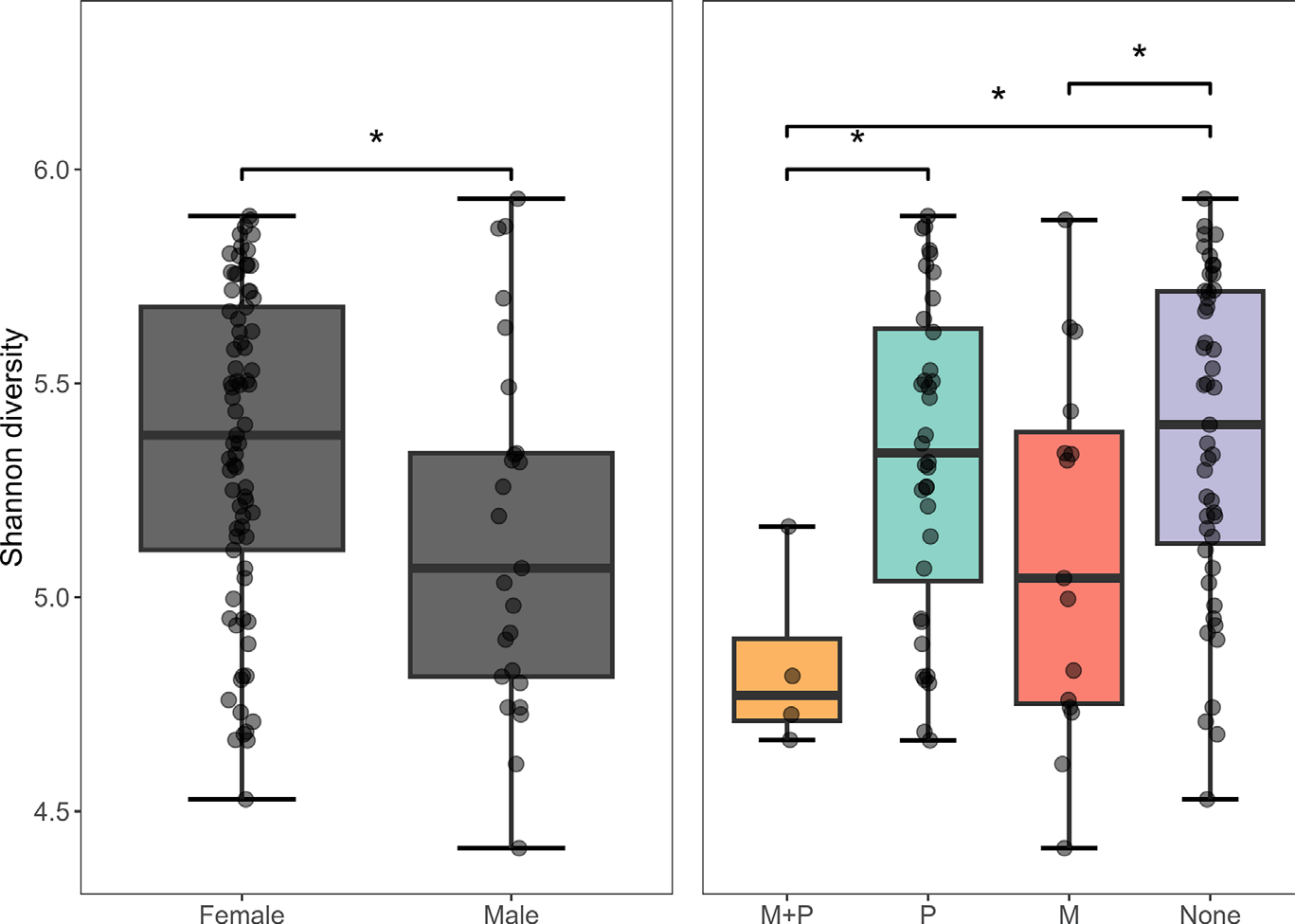
Alpha diversity (Shannon) of the microbiota of pooled caecal samples from 102 Norwegian turkey flocks, stratified on host sex and exposure groups: M+P, monensin and phenoxymethylpenicillin (PcV); M, monensin only; P, PcV only; and None, neither monensin nor PcV. Significant differences are indicated with brackets and an asterisk (*P*<0.05).

Exposure group explained 5.0% of the calculated Shannon diversity (*P*<0.01), while the host sex and farm ID explained 4.2% and 1.6%, respectively (*P*<0.05). Post hoc Tukey tests indicated that the alpha diversity is lower among the samples from turkey males compared with females (*P*<0.05, Fig. 2). A lower alpha diversity was also detected in the M and M+P groups compared with the ‘None’ group (*P*<0.05), as well as the M+P group compared with the P group (*P*<0.05). No difference in alpha diversity was detected at the farm level.

#### Beta diversity

**Fig. 3.**
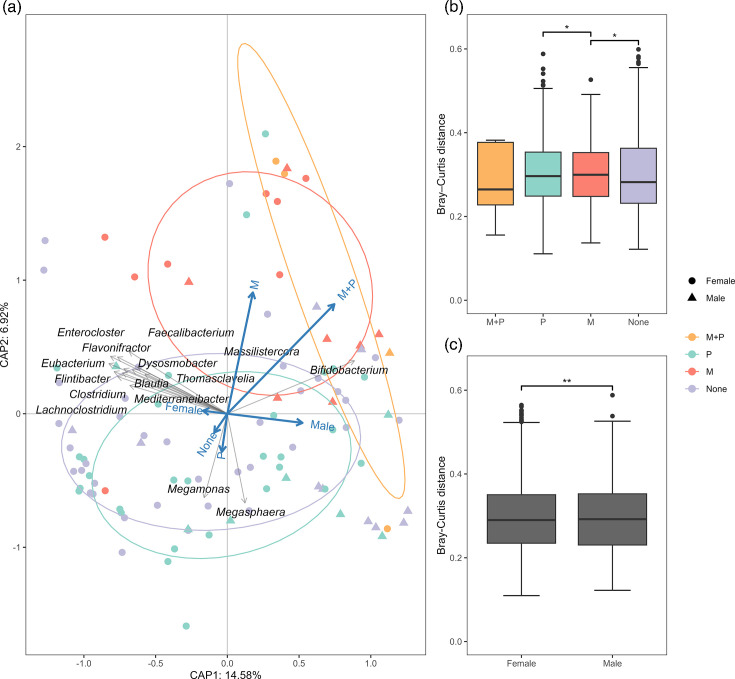
Beta diversity of the bacterial microbiota from 102 flock-wise pooled turkey caecal samples. (**a**) RDA of Bray–Curtis distances at the genus level and the explanatory variables. Grey arrows indicate the top 15 genera with the largest *R*-value. Blue arrows indicate the direction of the explanatory variables, i.e. the exposure groups: M+P, monensin and PcV; M, monensin only; P, PcV only; and None, neither monensin nor PcV. (b, c) Boxplots of within-group Bray–Curtis distances. Significant differences identified with pairwise PERMANOVA are denoted with asterisks, *P<0.05 and **P<0.01.

Sex of the birds had the biggest impact on the calculated Bray–Curtis dissimilarity, explaining 12.6% of the variability observed (*P*<0.01), while the exposure group and farm ID only explained 4.0% and 1.6%, respectively (*P*<0.01). No dispersion tests were significant. The pairwise PERMANOVA revealed a significant difference in beta diversity between the groups M and ‘None’ and between P and M, as well as between the sexes (*P*<0.05, Fig. 3b, c).

#### Constrained analysis

The RDA indicated that sex of the birds, sample type and farm ID were all significant explanatory variables for the bacterial microbiota composition (*P*<0.01), explaining a total of 17.8% of the observed variability (Fig. 3a). The axes CAP1 and CAP2 explained 15.1% and 7.0% of the variability, respectively (*P*<0.05). The genus *Bifidobacterium* was negatively correlated with samples from the ‘None’ and P groups and positively correlated with the M and M+P groups, while the opposite was observed for the *Megamonas* and *Megasphaera* genera. Further, several genera, such as *Clostridium*, *Faecalibacterium* and *Enterocloster*, were negatively correlated with samples from turkey males (Fig. 3a).

**Fig. 4.**
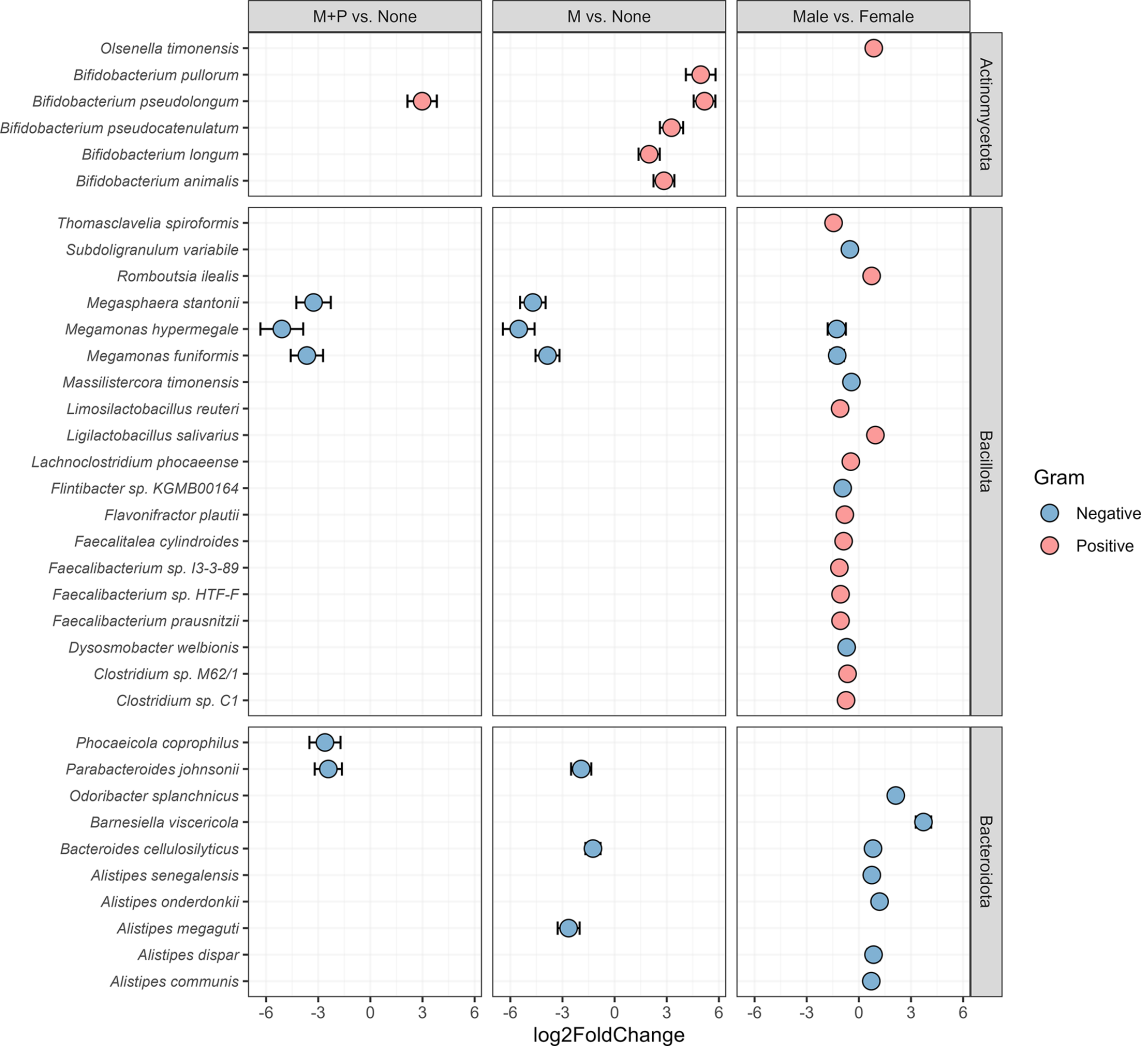
Taxa with significant differential abundance from the DESeq2 analysis (*P*≤0.05) of the bacterial microbiota from 102 flock-wise pooled turkey caecal samples. Only taxa with at least 1% relative abundance were included in the DESeq2 analysis. Each group was compared with the group that received no monensin or phenoxymethylpenicillin (PcV) treatment (denoted as ‘None’): P, PcV used; M+P, monensin and PcV used; and M, monensin used. The third column represents the comparison between the sexes of the sampled birds (male vs. female). All taxa are separated into phylum, listed on the right-hand side. The points are coloured based on the gram stain of that specific taxa, and error bars represent standard errors.

Differential abundance of taxa at the species level was investigated with DESeq2 (Fig. 4 and Table S1, available in the online Supplementary Material). For the male vs. female comparison, a total of 26 significant taxa were identified as differentially abundant. The top five species with the biggest fold change were *Barnesiella viscericola* (log2FC: 3.71, *P*_adj_<0.001), *Odoribacter splanchnicus* (log2FC: 2.12, *P*_adj_<0.001), *Thomasclavelia spiroformis* (log2FC: −1.45, *P*_adj_<0.001), *Megamonas hypermegale* (log2FC: −1.27, *P*=0.037) and *Megamonas funiformis* (log2FC: −1.25, *P*_adj_=0.006). For the M vs. ‘None’ exposure groups, a total of 11 taxa were identified, and the top 5 were *Megamonas hypermegale* (log2FC: −5.51, *P*_adj_<0.001), *Bifidobacterium pseudolongum* (log2FC: 5.17, *P*_adj_<0.001), *Bifidobacterium pullorum* (log2FC: 4.95, *P*_adj_<0.001), *Megasphaera stantonii* (log2FC: −4.70, *P*_adj_<0.001) and *Megamonas funiformis* (log2FC: −3.86, *P*_adj_<0.001). Six significant taxa were differentially abundant between the M+P and ‘None’ exposure groups, namely *Megamonas hypermegale* (log2FC: −5.08, *P*_adj_=0.002), *Megamonas funiformis* (log2FC: −3.65, *P*_adj_=0.003), *Megasphaera stantonii* (log2FC: −3.27, *P*_adj_=0.014), *Bifidobacterium pseudolongum* (log2FC: 2.98, *P*_adj_=0.008), *Phocaeicola coprophilus* (log2FC: −2.6, *P*_adj_=0.034) and *Parabacteroides johnsonii* (log2FC: −2.42, *P*_adj_=0.024). In the monensin exposure groups, all significant taxa with a negative log2FC were gram-negative, while the significant taxa with a positive log2FC were gram-positive. No significant hits were identified for the P vs. ‘None’ exposure group comparison.

Within the Farm 1 samples (*n*=3), a total of 42 taxa were identified that were differentially abundant compared with samples from 27 other farms. This included taxa such as *Megamonas hypermegale* (log2FC: 7.49, *P*<0.001), *Bifidobacterium pseudocatenulatum* (log2FC: 7.18, *P*<0.001), *Bifidobacterium pseudolongum* (log2FC: 6.13, *P*<0.001) and *Parabacteroides goldsteinii* (log2FC: 5.90, *P*<0.001) (see Table S1 for the full list of significant taxa).

## Discussion

In this study, the microbiota of 102 flock-wise pooled caecal samples from healthy turkey flocks sampled at slaughter were investigated in relation to prophylactic monensin use and antibiotic treatment. The results indicate a substantial increase in the abundance of *Bifidobacterium* spp. among the samples from turkeys fed monensin-supplemented feed, accompanied by a significant reduction in overall alpha diversity.

Treatment with PcV early in the rearing period did not seem to alter the microbiota composition or diversity at the time of slaughter, as indicated by the similarity between this group and the one not exposed to antibiotics or monensin (i.e. the ‘None’ group). On the other hand, the use of monensin seems to cause long-term alteration of the microbiota composition by reducing the overall diversity and by increasing the abundance of different species within the genus *Bifidobacterium*. The different results observed for PcV vs. monensin may be explained by the prolonged use of monensin over several weeks, as compared with a singular treatment with PcV over a shorter time span, thus giving the microbiota time to recover. However, the age at the time of treatment varies depending on the onset of the disease; treatment for GEU is typically administered at an earlier age compared with necrotic enteritis. Longitudinal sampling, considering both age and PcV treatment and/or monensin use, is needed to prove the hypothesized long-term alterations of monensin use on the microbial composition. Another aspect to consider is the differing antimicrobial effect of PcV compared with monensin and the resulting difference in affected bacteria, e.g. presence of resistance genes and other mechanisms of defence. This is, however, not taken into account within this study.

*Bifidobacterium* is a genus within the phylum *Actinomycetota* and is frequently found in the gut of humans [23,24] and in poultry [25]. Species within the genus are generally regarded as beneficial [24] and are frequently used in probiotic supplements. Studies have indicated that the administration of a probiotic with *Bifidobacterium,* more specifically *Bifidobacterium breve*, reduces the incidence of necrotizing enterocolitis and reduces the abundance of *C. perfringens* in humans [26,27]. In chicken, *Bifidobacterium bifidum* administered as a postbiotic has been found to reduce the abundance of harmful bacteria [28]. *Bifidobacterium breve* or *bifidum* was not detected in the current study. However, other *Bifidobacterium* species have been found to prevent infections in the gastrointestinal tract by competitive exclusion [29,30]. It is therefore possible that some, if not all, of the *Bifidobacterium* spp. detected in our study may exhibit similar characteristics. One might thus extrapolate that the increased abundance of *Bifidobacterium* within the monensin-exposed group may reduce the abundance of *Clostridia*, as evidenced by the slightly negative correlation in the RDA plot. It is generally accepted that a high diversity in the gut microbiota is beneficial. The overall decrease in diversity observed in the monensin-exposed flocks may therefore indicate a dysbiosis. However, it is unknown whether this decreased diversity is due to the competitive nature of *Bifidobacterium* spp. or directly due to the use of monensin. Further studies are needed to understand how the increased abundance of *Bifidobacterium* spp. and decreased overall diversity affect the health of the host in the presence of monensin use. Additionally, investigating the effect of monensin on other compartments of the gastrointestinal tract would be of interest, as natural differences in microbiota composition within each compartment could be affected differently by antibiotics and/or monensin.

*Megamonas* is an important propionate producer [31], and an association between spotty liver disease (SLD) in layer chickens and a low abundance of *Megamonas* has previously been identified [32]. The primary causative agent for SLD is thought to be *Campylobacter hepaticus*, and *Megamonas* genera have been found to cause a reduced relative abundance in the *C. hepaticus* challenged group compared with the unchallenged control group [31]. *Megasphaera* is a butyrate-producing organism that utilizes lactate as a precursor for butyrate [33,34] and was previously isolated from the caecum of healthy chicken [35]. A reduced abundance of *Megamonas* and *Megasphaera* in turkeys exposed to monensin contrasts with the general consideration that gram-negative bacteria are insensitive to monensin [36]. Similarly, cultures of *Megasphaera elsdenii* display monensin insensitivity *in vitro* [37,39], while no such studies to our knowledge exist for *Megamonas*. However, the observation aligns with a significant decrease in the abundance of *Megasphaera elsdenii* observed in monensin-treated dairy cows [40]. This discrepancy between *in vitro* and *in vivo* findings could suggest that the reduced abundance is a consequence of altered competition dynamics rather than a direct antibacterial activity of monensin. This hypothesis is further supported by the observation of an overall decrease in gram-negative taxa and an increase in gram-positive taxa in the monensin-exposed groups. A higher abundance of *Megasphaera* and *Megamonas* has previously been detected in male turkeys compared with female turkeys [41], based on 16S rRNA gene sequencing. Interestingly, the opposite was observed in the current study. However, it is difficult to compare studies that utilize different sequencing methodologies.

Several differences in microbiota composition were attributed to the sex of the sampled bird. However, sex and age of the birds (days) were identified as collinear terms in our analysis. Thus, it is highly likely that the microbiota differences are due to the age difference between males and females, where males live ~40 days longer than females. This longer life span may give rise to a slightly different microbiota composition, which in turn might affect how prophylactic use of monensin and/or PcV treatment affects the microbiota. Interestingly, the slightly increased abundance of *Clostridium* species in the samples from female turkeys may indicate that this genus plays a role in the development of the intestinal microbiota, which is in concordance with results from a study on broilers [11].

Differences between farms were evident in the DESeq2 analysis, especially for Farm 1. This may indicate that Farm 1 is an outlier compared with the other farms. Since there are likely to be differing management practices among the farms, this observed difference was not unexpected and could affect the results even though ‘farm’ was included as a covariate in our analysis. However, the DESeq2 analysis is robust, and the same results were identified when excluding Farm 1 from the analysis. Differences in management practices may include variation in cleaning and disinfection routines, which are done between each production cycle. Such differences are likely to have an impact on the in-house environmental microbiota and thereby on the gut microbiota of the birds.

## Conclusions

Our results indicate that the use of monensin seems to promote the abundance of *Bifidobacterium* spp. in the turkey caecum, while decreasing the abundance of *Megamonas* and *Megasphaera* species. Additionally, the overall diversity was decreased in the monensin-exposed birds, which may indicate dysbiosis. In contrast, treatment with PcV early in the life cycle of the animals does not seem to cause long-term changes in the caecal microbiota composition. Taken together, the results indicate that the use of monensin may be beneficial for the gut microbiota due to an increase in beneficial *Bifidobacterium* spp. However, further studies are needed to investigate the effects of a decreased abundance of *Bifidobacterium* spp. and increased gut microbiota diversity in turkey in the absence of monensin use.

## Supplementary material

10.1099/mgen.0.001466 Uncited Table S1.

## Abbreviations

GEU: gizzard erosion and ulceration syndrome
NVI: Norwegian Veterinary Institute
PcV: penicillin V
PERMANOVA: permutational multivariate ANOVA
RDA: redundancy analysis
SLD: spotty liver disease
